# A Novel Technique for Sterilization Using a Power Self-Regulated Single-Mode Microwave Cavity

**DOI:** 10.3390/s17061309

**Published:** 2017-06-07

**Authors:** Juan D. Reverte-Ors, Juan L. Pedreño-Molina, Pablo S. Fernández, Antonio J. Lozano-Guerrero, Paula M. Periago, Alejandro Díaz-Morcillo

**Affiliations:** 1 Department of Information and Communication Technologies, Universidad Politécnica de Cartagena, Plaza del Hospital, 1, 30202 Cartagena (Murcia), Spain; juand.reverte@upct.es (J.D.R.-O.); antonio.lozano@upct.es (A.J.L.-G.); alejandro.diaz@upct.es (A.D.-M.); 2 Department of Food Engineering and Agricultural Equipment, Universidad Politécnica de Cartagena, Paseo Alfonso XIII, 48, 30203 Cartagena (Murcia), Spain; pablo.fernandez@upct.es (P.S.F.); paula.periago@upct.es (P.M.P.)

**Keywords:** biosensors, microbiological food safety, microwave cavity, power and temperature control, sterilization, animal by-products

## Abstract

In this paper, a novel technique to achieve precise temperatures in food sterilization has been proposed. An accurate temperature profile is needed in order to reach a commitment between the total removal of pathogens inside the product and the preservation of nutritional and organoleptic characteristics. The minimal variation of the target temperature in the sample by means of a monitoring and control software platform, allowing temperature stabilization over 100 °C, is the main goal of this work. A cylindrical microwave oven, under pressure conditions and continuous control of the microwave supply power as function of the final temperature inside the sample, has been designed and developed with conditions of single-mode resonance. The uniform heating in the product is achieved by means of sample movement and the self-regulated power control using the measured temperature. Finally, for testing the sterilization of food with this technology, specific biological validation based on *Bacillus cereus* as a biosensor of heat inactivation has been incorporated as a distribution along the sample in the experimental process to measure the colony-forming units (CFUs) for different food samples (laboratory medium, soup, or fish-based animal by-products). The obtained results allow the validation of this new technology for food sterilization with precise control of the microwave system to ensure the uniform elimination of pathogens using high temperatures.

## 1. Introduction

Conventional sterilization procedures for packaged foods are based on retort treatments which are subjected to high pressure and temperatures in excess of 100 °C to guarantee their safety. To ensure the absence of pathogenic microorganisms, as well as toxic substances for the consumer, it is necessary to establish the food safety of the final product that will be consumed. Specifically, 0.1 mg of food contaminated with botulinum toxin can cause botulism, since ingestion of doses as low as 30–100 ng can be even lethal to the consumer [1], so the requirement for uniformity in heating to ensure microbial inactivation must be maximized [2].

Treatment of food by-products (waste) is also of upmost importance in order to efficiently reduce their environmental impact. The use of microwaves for sterilization processes can be, in many cases, very restrictive. In addition, the pH value in the food significantly influences the sterilization process. The problems of sterilization or pasteurization of foods with microwaves are well known in the industrial field [3,4].

Although microwave-sterilized products present a lower loss of nutrients compared to those processed with conventional methods, if the process is not well adapted or suitable to the product the loss of quality can be greater with microwave treatment in some cases. The problem that exists here is the difficulty of obtaining uniform heating throughout the product using microwave technology [5,6]. In food sterilization or waste processes, this uniform control is the key for obtaining a safe product while maintaining its nutritional quality. This is the main obstacle in the use of microwaves, as well as the definition of the process criteria for microorganisms, such as *Clostridium perfringens*, *Salmonella*, and *Enterobacteriaceae*.

The non-uniform heating of the product is determined by two fundamental reasons. First, although the exponential decay of dissipated microwave energy is only valid for the model of a plane wave hitting a semi-infinite plane product, it is a good approximation when the material in the cavity is homogeneous and lossy [7], as is the case for the products analyzed in this paper. Thus, in the case of products with high losses, such as biological ones, the wave is rapidly attenuated. As a consequence of this fact, laminar products with a thickness around 1–2 cm must be formed if a good uniformity of heating is desired.

On the other hand, the electric field has a high spatial variability inside a resonant cavity, which results in a non-homogeneous heating, due to the direct relation between electric field strength and generated heat. This is true for both single-mode and multi-mode cavities. In a multi-mode cavity, the resulting electric field is a linear combination of the different resonant modes or waves excited in the cavity. Attempts to improve this spatial uniformity have traditionally been based on mechanisms of product movement (turntable, conveyor belt) or mode stirrers [3] (which produce a time variation of the electric field pattern in the cavity). With these two solutions, it is possible to obtain a dissipated energy, time-averaged, similar in all of the points of the sample, but it does not necessarily guarantee a uniformity of temperature as required in sterilization processes.

In recent years, the use of microwave technology at 915 MHz [8], compared to the more developed and traditional 2.45 GHz, has been studied for these cases. Although the use of lower frequencies undoubtedly improves the uniformity of the electric field inside the applicator, allowing a greater penetration of the wave in the material, this technology is much more expensive, especially in the microwave sources, which makes it difficult to transfer to the industry.

At present, current research focused on uniform electric field distributions on the surface of the treated product is based on multimode applicators, which limits the application of microwaves to a large number of industrial heating processes, including sterilization processes. Recent techniques in multimode cavity simulation platforms are based on low-temperature heating processes using multiple ports with different phase feeds [9], optimization by parameter variation with microwave CAD (computer aided design) technology [10,11], determination of the optimal point of a sample to minimize the deviation of a heating pattern [12], or the use of stirrers [7]. However, when a sterilization process with temperatures over 100 °C is required, microwave heating conditions to minimize the expected temperature deviation must be controlled.

Recent studies have demonstrated better results for food pasteurization or sterilization in terms of retention of nutrient quality [13] and microbial elimination [14], when microwave technology is used in comparison with conventional autoclaving. Specific developments have also been simulated for sterilization processes [15]. In [16] a very high microwave power in a cylindrical cavity was employed to measure the effect of sterilization in a specific food sample. However, although in this study the sterilization process is made by using microwave systems, the uniform temperature profile throughout the sample is not confirmed.

In this work, a real installation based on a single-mode pressurized cavity, online data acquisition system, control software, and a monitoring system has been designed and implemented for modelling the food sterilization process.

Another limitation to ensure real uniformity in sterilization processes is the absence of microwave-adapted biological methods to determine the degree of variability in heating achieved based on microbial inactivation at each point of the food. This implies the difficulty of using existing biosensors that can efficiently measure the effectiveness of the heating process and its variability.

With reference to biological sensors, another objective of this work is to obtain and verify, by measuring CFUs as a parameter, a microwave device with high volumetric heating uniformity in temperature processes above 100 °C for sterilization of food and animal waste. For this, a specific geometry of the applicator has been designed, under pressure conditions, with internal movement mechanisms and with biosensors. The system is controlled online by MATLAB^®^ software (Natick, MA, USA) that continuously determines the profile of the time-power curve to minimize the final deviation for the target temperature and ensure the pathogen inactivation.

On the other hand, the development of biosensors are necessary to establish the food safety of the final whole product, with accurate information of the main points that are representative of the sample, which assures the absence of pathogenic microorganisms and toxic substances for consumption [17]. Microbiological biosensors have been used to validate conventional heat sterilization processes in which colder points are known [18]. In the case of microwave heating, uncertainty about cold spots is the main challenge to ensure the safety of food or treated products and, therefore, to characterize a suitable microorganism that can evaluate uniformity in different substrates was a key step.

## 2. Materials, Methods, and Experimental Set Up

### 2.1. Materials

The electromagnetic response of different samples of soups and fish wastes have been studied and electric permittivity was characterized as a function of temperature at the frequency of around 2.45 GHz by means of a commercial dielectrometer from ITACA (Mod. Dialkitv, Valencia, Spain). This measurement is based on a resonant method, which obtains the value of the electric permittivity from the shift of the resonant frequency and the decrease of the quality factor when a dielectric material is placed in a resonant cavity [19]. The equipment allows the introduction of a standard 8 mL polypropylene vial (12 mm diameter and 75 mm length). Resonant methods are described in detail in [20].

From the permittivity measurements, the single-mode resonant cavity has been designed to perform small-sample pressure (test tube) heating tests in such a way as to guarantee uniformity of heating in the sample, and including temperature sensors that monitor the treatment of samples with a wide range of permittivity. A fiber optic Opsens (EQ013 TEMPSENS) (Quebec, QC, Canada) temperature measurement device has been introduced in the sample to obtain the temperature of the sample in real-time. The fiber optic temperature sensor has been placed in the geometrical center of the sample. Since a high uniformity is expected due to the single-mode operation, the small size of the sample, and its stir, this position becomes a meaningful indicator far away from the surface of the sample where the gradient with the outer temperatures may hinder a correct measurement.

**Figure 1:**
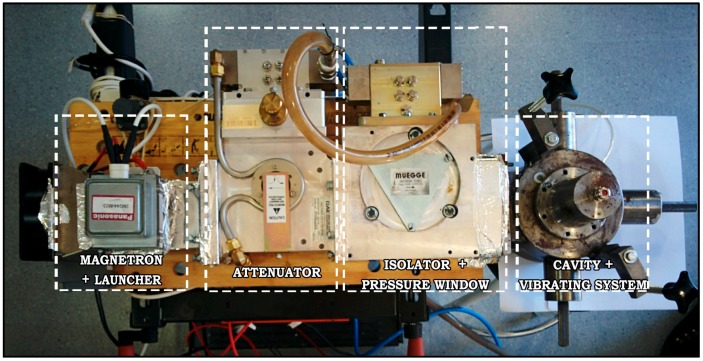
Experimental microwave single-mode cylindrical cavity prototype to obtain precise temperature profiles for sterilization purposes.

As Figure 1 shows, the developed prototype is formed by the Muegge (Mod. MX1000C-112KL, Reichelsheim, Germany) power supply, offering a precise continuous control of the heating power, which has been programmed using MATLAB^®^; the 1 kW standard component (Panasonic Mod. 2M244-M23, Osaka, Japan) magnetron, which transforms the electrical energy into microwave electromagnetic energy; the launcher to transmit the energy of the magnetron to the heating system; the (GAE GA1118, Reichelsheim, Germany) attenuator to adapt the microwave power to the 3 mL food samples; the isolator, implemented using a circulator Muegge (Mod. MM1003A 210EC), in order to protect the magnetron by deviating the reflected microwave energy to be dissipated by means of a water load, with a connection to include an incoming power detector; and, finally, the resonant cylindrical cavity, whose height is 4.34 cm, and diameter is 13.4 cm.

The excited mode to heat the sample is the transversal magnetic TM_010_. This mode shows invariance with the sample height and a vertical component for the electric field.

**Figure 2:**
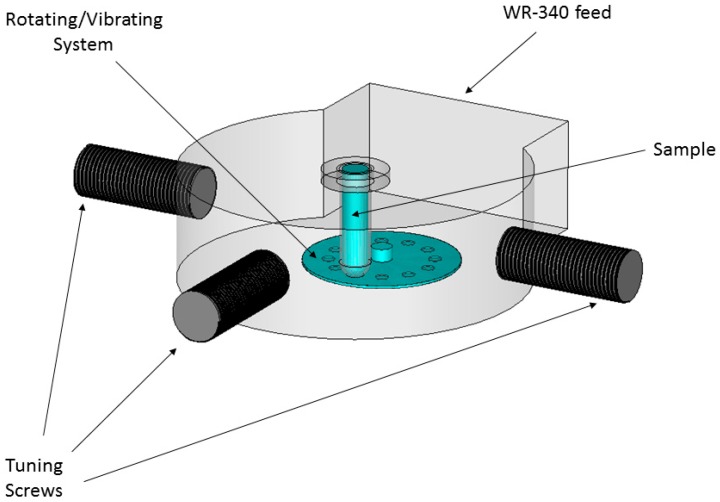
Designed cavity, including the tuning and vibration systems.

The designed cavity, shown in Figure 2, includes a tuning system to adapt the load to be heated, a vibration system in order to obtain a greater heating uniformity, and the pressure system to reach higher temperatures over 100 °C. The tuning system has been carried out using stubs or screws, which allow the modification of the frequency of resonance of the cavity and to adapt it as much as possible for a wide range of materials. The vibratory system consists of a PTFE plate with holes in its rotating surface driven by a motor, moving the sample up and down with a frequency of approximately five times per second. This system has been designed and built to improve the uniformity of the temperature.

The opening and closing system has been designed to be able to safely close the cavity when working under pressure up to 5 atm, and to be able to open it quickly to remove the sample.

The system is pressurized by the combination of three designs: O-rings in screws and the cover, septum in the fiber area temperature sensors, and a pressure window in the connection section of the power feeding system with the resonant cavity.

### 2.2. Software Implementation

**Figure 3:**
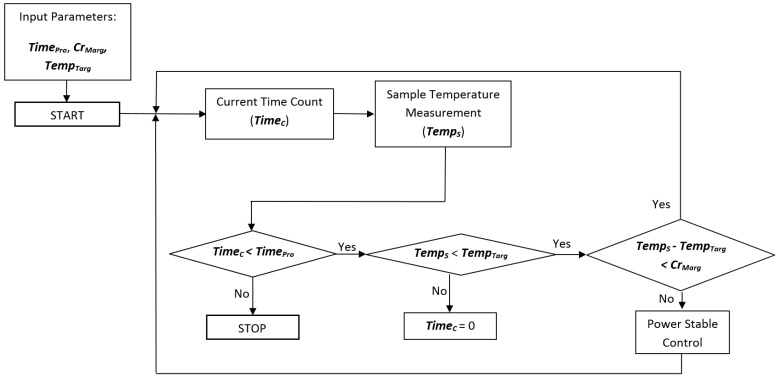
Flow diagram of the adaptive procedure obtaining the desired temperature during the microwave treatment time, by considering processing time (*Time_Pro_*), current time (*Time_C_*), critical margin (*Cr_Marg_*), sample temperature (*Temp_S_*), and target temperature (*Temp_Targ_*).

In order to control the entire microwave heating system, a platform including the information from the sensors, actuation, and process monitoring by means of adaptive control software has been developed. This control allows the supplied power to be adjusted in such a way that the sample maintains the desired temperature as long as needed. A flow diagram of the complete procedure is shown in Figure 3.

**Figure 4:**
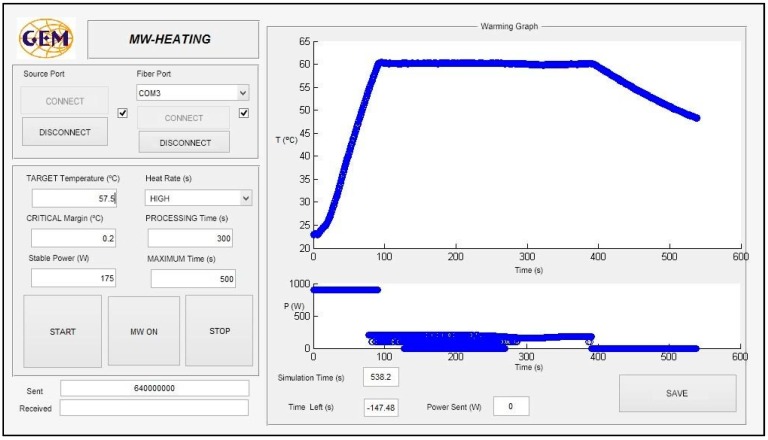
Interface of the developed home-made GUI to control the developed system.

For the sterilization process, heating over 100 °C must be carried out. For this purpose, a compressed air system is connected to the cavity in order to regulate the pressure, using the pressure gauge. The critical margin is defined as the threshold over the difference between the current temperature and the final target for the sample. While the difference between the target temperature (*Temp_Targ_*) and the current temperature for the sample (*Temp_s_*) does not overcome the critical margin (*Cr_Marg_*) that is initially established, the power continues at the highest value, in order to reach the objective temperature with minimal time, avoiding overshoots. When this value is reached, the system autonomously decreases the power to enter the maintenance phase in which the stabilization power is adjusted as a function of the sample temperature. This is a fine adjustment of the power in order to maintain the sample temperature as constant as possible inside the defined initial threshold. A MATLAB^®^ graphical user interface (GUI) has been developed to control and monitor the process, as Figure 4 shows. It allows reporting of the real-time temperature and incident power profiles.

As can be observed, to achieve the desired behavior for the microwave sterilization process, different parameters must be selected from the interface software, as shown in Figure 4. The heating rate allows the selection of a certain percentage over the maximum power of the microwave supply. The main objective of including the critical margin is to adaptively establish the time in which the software system remains in the fine tuning phase, in order to avoid large overshoots, as well as to avoid overheating of the sample to a high temperature before being stabilized.

### 2.3. Microbial Determinations and Food Substrates

A highly heat-resistant, spore forming microorganism, *Bacillus cereus*, was selected as an indicator, because it is a common contaminant and foodborne pathogen of different foods and in its sporulated stage it presents high resistance to heat. The following food substrates (or laboratory media) were selected for the experiments: brain heart infusion broth as the control; vegetable puree as food, and a fish-based animal by-product (F-ABP), obtained from seabass homogenate.

The microorganism was homogenized in the different substrates tested at a known concentration, and exposed to different heating times and temperatures in the microwave installation previously described. Samples were cooled, diluted (ten-fold) in sterile peptone water, and plated in Petri dishes, using brain heart infusion agar as the recovery medium. Once the medium solidified, plates were incubated at 37 °C for 48 h and the resulting colonies were counted. Log_10_ counts of survivors (expressed as colony forming units, CFUs) were plotted against the exposure time for each temperature and inactivation kinetics were established.

## 3. Results and Discussion

### 3.1. Permittivity Characterization

The relative electric permittivity of the material is represented by $\varepsilon^{*}=\varepsilon^{\prime }+j\;\varepsilon^{\prime \prime }$ and describes the dielectric properties affecting the reflection and attenuation of the electromagnetic field energy in the material, $\varepsilon^{\prime }$ being the dielectric constant and $\varepsilon^{\prime \prime }$ being the material loss factor.

**Figure 5:**
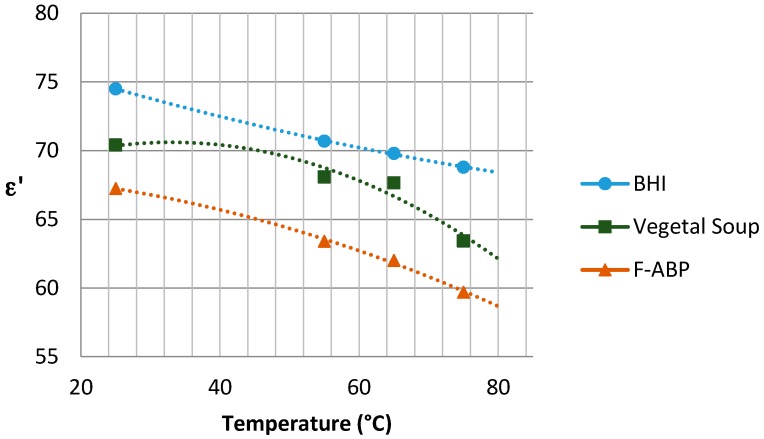
Dielectric constant for the samples under study near 2.45 GHz.

**Figure 6:**
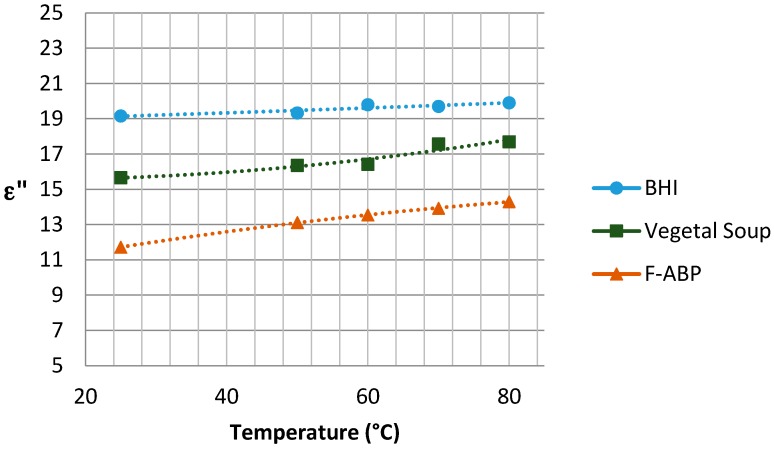
Loss factor for the samples under study near 2.45 GHz.

The graphs with the results concerning the dielectric constant (which defines the degree of polarization of the material to be subjected to an electric field) and the loss factor (which indicates the capacity of dissipation of electric energy within the material, demonstrated as heat [21]) are shown in Figure 5 and Figure 6, respectively, depending on the temperature reached in each test. Since the Dielkitv equipment does not allow pressurized measurements, the dielectric characterization of the products was limited under 100 °C. The selected materials are brain heart infusion broth (BHI), fish-based animal by-products (F-ABP), and vegetable soup, which consists of 30% vegetables, where the main ingredients are carrots, potato, celery, onions, tomato, turnip, pumpkin, corn starch, and dehydrated milk.

All of the samples presented a density close to 1 due to their high water content, although they differed in their lipid content. The mass used was in the range of 3 to 10 g, as this is a representative of the size used in food studies.

The resonant frequencies of an empty cavity differ from the ones of a fully-loaded cavity proportionally to a factor 1/√$\varepsilon^{\prime }$. The perturbational theory then indicates that a shift in the resonant frequency is directly related to a deviation in the dielectric constant of an inserted sample, but the tuning system allows the modification of the cavity geometry in order to keep the resonant frequency at 2.45 GHz, i.e., the operation frequency of the magnetron source. Thus, both the cavity dimensions and the screws were designed in CST Microwave Studio to be able to tune for a wide range of materials.

Although the behavior of the dielectric properties corresponding to the measured samples is almost linear, an adjustment based on second-order polynomial regression has been superposed to adequately show the trend for $\varepsilon^{\prime }$ and $\varepsilon^{\prime \prime }$.

### 3.2. Resonance

**Figure 7:**
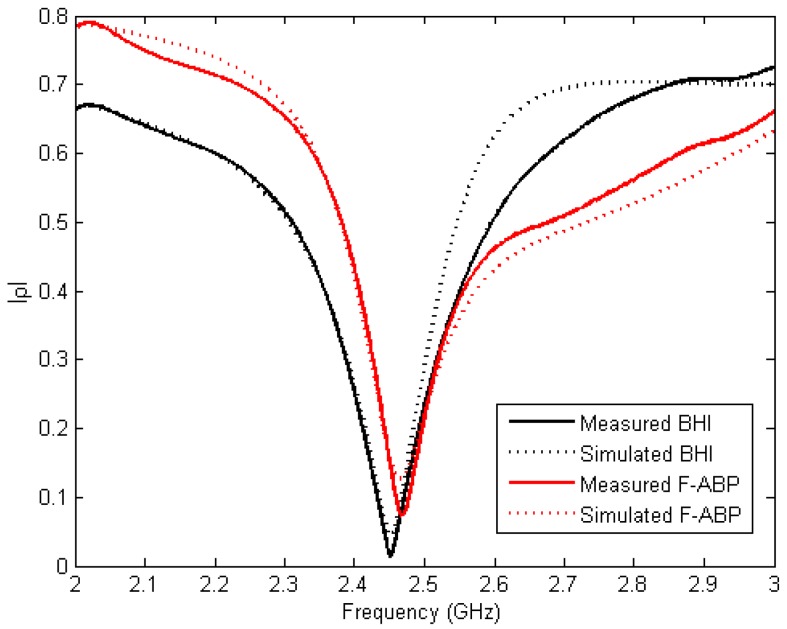
Measured and simulated reflection coefficient for BHI and F-ABP.

The reflection coefficient of BHI and F-ABP are shown in Figure 7. Measurements have been carried out directly by measuring the cavity depicted in Figure 2 with the corresponding samples. A Rohde and Schwarz Vector Network Analyzer ZVA 67 (München, Germany) has been used for the measurements with a WR-340 calibration kit. Simulated results have been obtained using the developed model in CST Microwave Studio (Darmstadt, Germany) with the measured values of permittivity obtained from Figure 5 and Figure 6 at room temperature. Slight differences may be due to inaccuracies in the CAD model or in the permittivity model in the whole frequency range of 2–3 GHz. The loaded system shows a clear resonance at frequencies near 2.45 GHz and this frequency can be easily reached using the tuning screws.

### 3.3. Temperature Profiles

**Figure 8:**
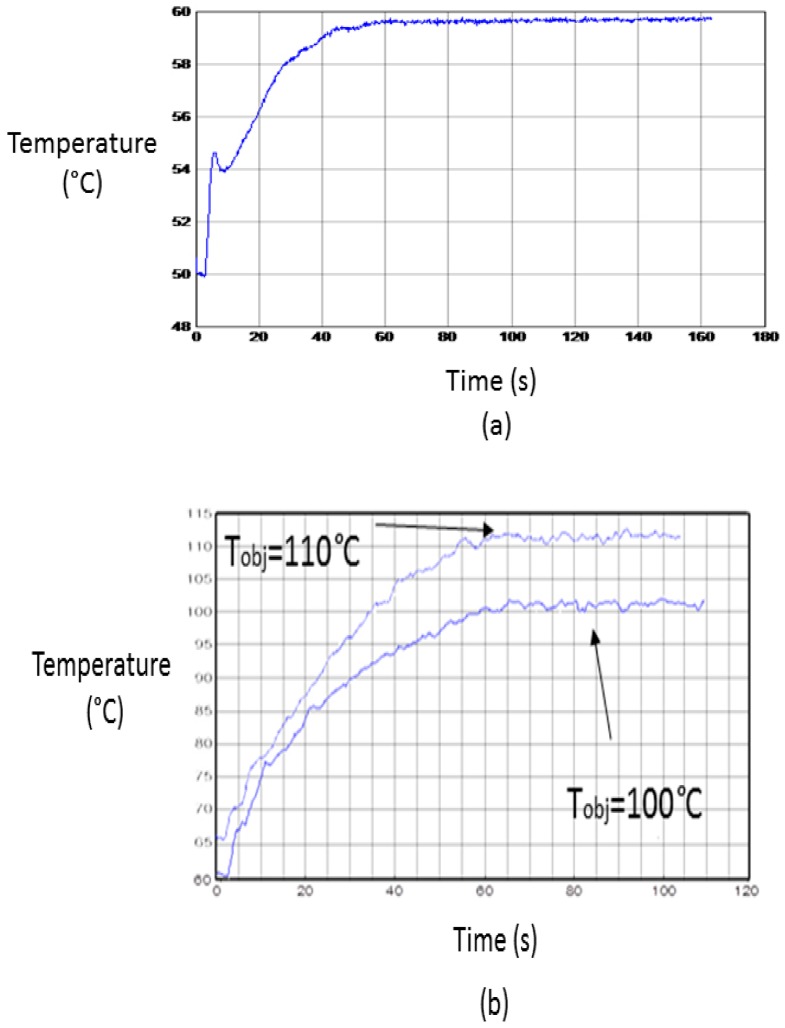
Temperature profiles of developed experiments. (**a**) Low-temperature process; and (**b**) high-temperature process.

Table 1 shows conditions for some of the experiments carried out. Under these conditions, the temperature profiles drawn in Figure 8 have been obtained. A 60 °C target temperature has been reached with no need of the pressure system. An error of −0.5% with a deviation of ±0.1 °C has been obtained. When the pressure system has been activated two examples for 100 °C and 110 °C have been reported. For these cases, errors of 1.7% and 2.2% with deviations of ±1.0 °C and ±1.1 °C were obtained, respectively.

The peak shown in Figure 8a corresponds to the behavior of the power control system when trying to follow a fast increment of temperature, the occurrence of small overshoots being frequent. The fit of the temperature profile to the required one allows the validation of the stability for the implemented microwave heating system. When a slower temperature increase is required (Figure 8b), the peaks are usually very small.

### 3.4. Heat Inactivation of Bacillus cereus

The number of survivors (expressed as log_10_ CFU/mL) at different heating times was plotted for each temperature tested. Although some survivor curves presented tailing and shoulders (especially when several log cycles of inactivation were achieved), since correlation coefficients were higher than 0.9 it was considered that, in general, they followed a first-order kinetic, although some curves could also be fitted with models considering shoulders and tailing. A non-uniform temperature would lead to a lower inactivation rate or to a deviation from linearity (tailing), so as neither were observed, it was concluded that homogeneous heating took place. The log-linear primary inactivation model considers that the microbial count, *N*, decreases exponentially with time, *t*. The time required for one decimal logarithmic reduction under constant temperature, *T*, is defined in Equation (1) by *D(T)*, and it is considered a first-order kinetic rate:

(1) $$\frac{d\log_{10}N}{dt}=-\frac{1}{D\left(T\right)}$$

The microwave power that is transformed in heat [22] can be obtained from Equation (2):

(2) $$P=2\pi f\varepsilon_{0}\varepsilon^{\prime \prime }{\left|E_{rms}\right|}^{2}$$

where $\varepsilon_{0}=8.8542\times 10^{-12}$ F/m is the free space electric permittivity, f(Hz) is the operation frequency, and $E_{rms}$(V/m) is the root mean square of the electric field. From this relationship, it can be deduced that the higher the electric field strength and loss factor the higher power values obtained will be. This dissipated power generates a heat *Q_gen_* in the material, which, following the heat Equation (3), produces an increment of temperature and, consequently, a bacteriological inactivation is observed,

(3) $$\rho c_{p}\frac{\partial T}{\partial t}=k_{T}\nabla^{2}T+Q_{gen}$$

where *T* is the temperature, *t* the time, *k_T_* is the thermal conductivity, *ρ* the density, and *c_p_* the specific heat of the material. Nevertheless, in biological products a high loss factor value is normally related to a high value in the dielectric constant, and this hinders the wave penetration inside the sample, decreasing the electric field strength.

**Figure 9:**
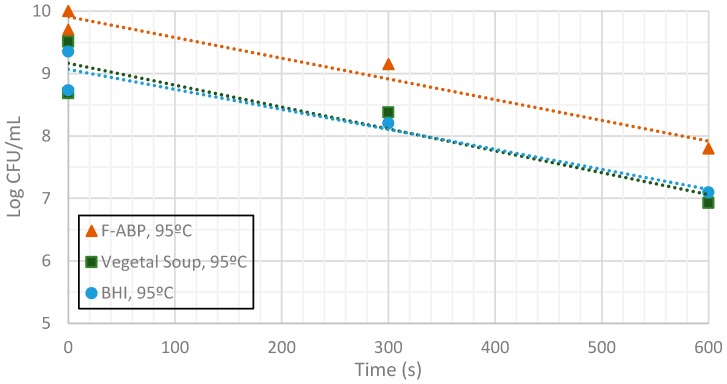
Measurement of CFUs in the substrates indicated at a target temperature of 95 °C.

**Figure 10:**
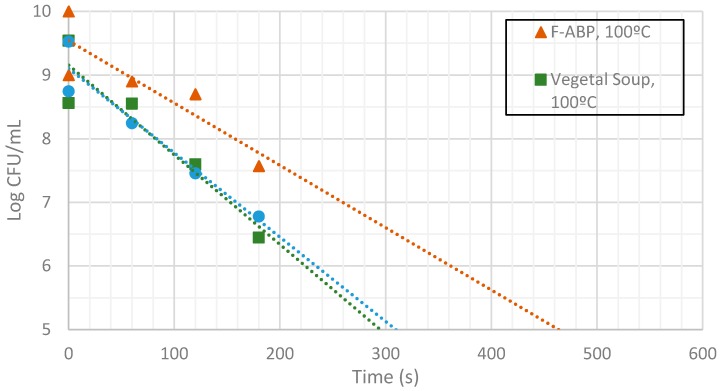
Measurement of CFUs in the substrates indicated at a target temperature of 100 °C.

**Figure 11:**
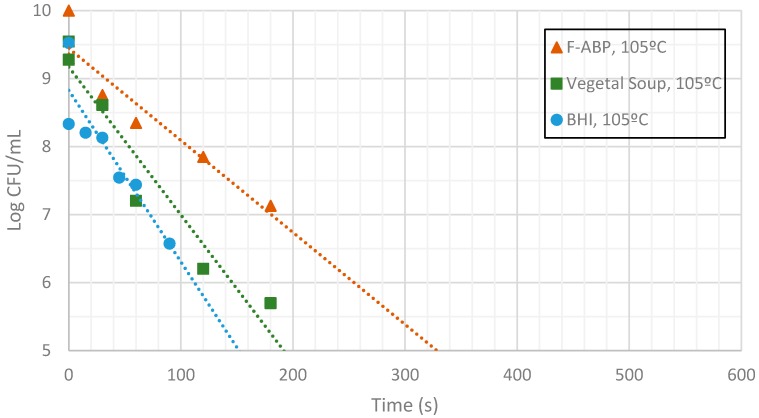
Measurement of CFUs in the substrates indicated at a target temperature of 105 °C.

Figure 9, Figure 10 and Figure 11 show the evolution of the CFUs of *Bacillus cereus* spores at different heating times. A log-linear interpolation is provided and clearly predicts the evolution of the number of survivors. Clearly, higher temperatures provide a better sterilization in terms of CFU showing a steeper slope.

Inactivation shows, as expected, a temperature dependency: the higher the target temperature, the faster inactivation occurred (Figure 9, Figure 10 and Figure 11). This indicates that there is an accurate temperature control in the microwave system. At all of the temperatures studied (95 °C, 100 °C and 105 °C), inactivation rate is higher in BHI and lower in F-ABP. There is a high log-linear correlation coefficient for all the survival curves obtained (always higher than 0.9) and counts observed were not lower than those obtained by conventional heating, which indicated that a homogeneous heating is obtained [23,24].

At 105 °C a fast inactivation is observed, with 3 log decimal reductions in less than 120 s in BHI, whereas to achieve the same inactivation in F-ABP more than 200 s are necessary, as Figure 11 shows. Vegetable soup required a time in between. Differences in heat inactivation can be explained by three main reasons:

- Higher loss factors lead to steeper temperature increase in the first heating stage, when a constant microwave power is applied to the cavity, since a higher loss factor yields a larger heat generation. Consequently, the target temperature is reached faster in the sample and, therefore, it is treated at the target temperature for a longer time. This rationale cannot be applied to the heating control stage, since there the power is controlled to keep the target temperature and, therefore, the heating generation is not directly related to the loss factor.
- Heat transfer by conduction inside the sample is higher with lower lipid content, since the thermal conductivity of the water, as a polar molecule, is higher than that of lipids, which are non-polar molecules.
- The presence of lipids can also have a protective effect on the spores of *Bacillus cereus* [25].

## 4. Conclusions

A single-mode cylindrical cavity has been designed jointly with a self-regulated power-supply system for achieving a selected temperature in order to study the sterilization of different samples. The correct operation of the designed cavity and the self-regulated system have been successfully verified. Parameters, such as the permittivity of the samples have been provided in a wide range of temperatures from 20 °C to 80 °C.

Accurately and uniformly enforcing a selected temperature in the volume of the sample with the developed microwave system has allowed a systematic study of the evolution in the bacterial counts of three different materials to be sterilized. This system can effectively ensure thorough and uniform microbial inactivation in foods.

Heat inactivation by microwave heating in different substrates (laboratory media and foods) has been tested using a foodborne pathogenic bacterium, *Bacillus cereus*, as a sensor. It shows a log-linear decline in all the conditions tested (temperatures between 95 °C and 105 °C) and a similar inactivation rate to conventional heating, indicating a homogenous temperature profile. Inactivation rate is higher in BHI and lower in F-ABP, which is related to the loss factor of each product and, ultimately, to the content of lipids.

As an alternative, a solid-state microwave generator could have been used. In that case, the power control is more accurate and quite easier; therefore, probably smaller variations around the goal temperature would have been obtained. However, solid-state microwave generators are very limited in power (as much as a few hundreds of watts) and are very expensive, which invalidates its use for high power applications, i.e., 1–100 kW. On the contrary, the analysis presented here deals with a magnetron source, which allows a scalable system.

Single-mode cavities, small sample and a vibratory system lead to uniform temperature profile along the material, and the main challenge is to keep the temperature constant with time, as shown. The next step is the design of multi-mode applicators in order to deal with larger samples at an industrial scale. In this case, future work is envisaged to reach a spatial uniformity of temperature that guarantees pasteurization or sterilization while keeping the nutritional and organoleptic properties.

## Figures and Tables

**Table 1:** Conditions for the experiments.

Process	Pressure	Time	Objective
Low temp.	No	100 s	60 °C
High temp.	Yes	50 s	100 °C
High temp.	Yes	50 s	110 °C

## References

[1] Brunt J., Plowman J., Gaskin D.J.H., Itchner M., Carter A.T., Peck M.W. (2014). Functional characterisation of germinant receptors in *Clostridium botulinum* and *Clostridium sporogenes* presents novel insights into spore germination systems. PLoS Pathog..

[2] Martínez-Lopez A., Rodrigo D., Fernández P.S., Pina-Pérez M.C., Sampedro F. (2012). Time-Temperature integrators for thermal process evaluation. Thermal Food Processing-New Technologies and Quality Issues.

[3] Stanley R.A., Petersen K., Regier M., Knoerzer K., Schubert H. (2017). Microwave-assisted pasteurization and sterilization—commercial perspective. The Microwave Processing of Foods.

[4] Bengtsson N.E., Ohlsson T. (1974). Microwave heating in the food industry. Proc. IEEE.

[5] Vadivambal R., Jayas D.S. (2010). Non-uniform temperature distribution during microwave heating of food materials—A review. Food Bioproc. Technol..

[6] Nguyen L.T., Choi W., Hyun Lee S., Jun S. (2013). Exploring the heating patterns of multiphase foods in a continuous flow, simultaneous microwave and ohmic combination heater. J. Food Eng..

[7] Plaza-Gonzalez P., Monzó-Cabrera J., Catalá-Civera J.M., Sánchez-Hernández D. (2005). Effect of mode-stirrer configurations on dielectric heating performance in multimode microwave applicators. IEEE Trans. Microw. Theory Tech..

[8] Tang Z., Mikhaylenko G., Liu F., Mah J.H., Pandit R., Younce F., Tang J. (2008). Microwave sterilization of sliced beef in gravy in 7-oz trays. J. Food Eng..

[9] Ejiri K., Tomizuka Y., Ichihara G., Sato H., Uchida Y., Ohno T., Uehara M. A study on uniform heating of food in microwave oven by using phase difference of power output from two ports. Proceedings of the Electromagnetic Research Symposium (PIERS).

[10] Cordes B.G., Eves E.E., Yakovlev V.V. Modeling-based minimization of time-to-uniformity microwave heating systems. Proceedings of the 11th AMPERE Conference on Microwave and High Frequency Heating.

[11] Gu X.W., Lin M., Yiqin S. (2010). Electromagnetic field optimisation procedure for the microwave oven. Int. J. Electron..

[12] Pedreño-Molina J.L., Monzó-Cabrera J., Catalá-Civera J.M. (2007). Sample movement optimization for uniform heating in microwave heating ovens. Int. J. RF Microw. Comput.-Aided Eng..

[13] Kothari V., Patadia M., Trivedi N. (2011). Microwave sterilized media supports better microbial growththan autoclaved media. Res. Biotechnol..

[14] Neetoo H., Chen H., Clark S., Jung S., Lamsal B. (2014). Alternative Food Processing Technologies. Food Processing: Principles and Applications.

[15] Resurreccion F.P., Luan D., Tang J., Liu F., Tang Z., Pedrow P.D., Cavalieri R. (2015). Effect of changes in microwave frequency on heating patterns of foods in a microwave assisted thermal sterilization system. J. Food Eng..

[16] Steed L.E., Troung V.D., Simunovic J., Sandeep K.P., Kumar P., Cartwright G.D., Swartzel K.R. (2008). Continuous Flow Microwave-Assisted Processing and Aseptic Packaging of Purple-Fleshed Sweet potato Purees. J. Food Sci..

[17] Zuijlen A.V., Periago P.M., Amézquita A., Palop A., Brul S., Fernández P.S. (2010). Characterization of *Bacillus sporothermodurans* IC4 spores; putative indicator microorganism for optimisation of thermal processes in food sterilization. Food Res. Int..

[18] Ocio M.J., Fernández P.S., Rodrigo M., Periago P.M., Martínez A. (1997). A time temperature integrator for particulated foods: Thermal process evaluation. Z. Lebensmittel Untersuchung und Forschung.

[19] Álvarez A., Fayos-Fernández J., Monzó-Cabrera J., Cocero M.J., Mato R.B. (2017). Measurement and correlation of the dielectric properties of a grape pomace extraction media. Effect of temperature and composition. J. Food Eng..

[20] Chen L.F., Ong C.K., Neo C.P., Varadan V.V., Varadan V.K. (2004). Microwave Electronics. Measurement and Material Characterization.

[21] Metaxas A.C., Meredith R.J. (1983). Industrial Microwave Heating.

[22] Datta A.K., Anatheswaran R.C., Marcel Dekker (2000). Handbook of Microwave Technology for Food Applications.

[23] Fernandez P.S., Ocio M.J., Rodrigo F., Rodrigo M., Martínez A. (1996). Mathematical model for the combined effect of temperature and pH on the thermal resistance of *Bacillus stearothermophilus* and *Clostridium sporogenes* spores. Int. J. Food Microbiol..

[24] Garre A., Fernandez P.S., Lindqvist R., Egea J.A. (2017). Bioinactivation: software for modelling dynamic microbial data. Food Res. Int..

[25] Esteban M.D., Huertas J.P., Fernández P.S., Palop A. (2013). Effect of the medium characteristics and the heating and cooling rates on the nonisothermal heat resistance of *Bacillus sporothermodurans* IC4 spores in buffers and food. Food Microbiol..

